# PRELIMINARY RESULTS OF THE CONFIDENCE FINANCIAL INTERVENTION FOR CAREGIVERS TO PERSONS LIVING WITH DEMENTIA

**DOI:** 10.1093/geroni/igad104.2000

**Published:** 2023-12-21

**Authors:** Kylie Meyer, Jaclene Zauszniewski, Lixin Song, Donna Benton

**Affiliations:** Case Western Reserve University, Cleveland, Ohio, United States; Case Western Reserve University, Cleveland, Ohio, United States; University of Texas Health Science Center at San Antonio, San Antonio, Texas, United States; University of Southern California, Los Angeles, California, United States

## Abstract

Family caregivers to persons living with dementia experience high out-of-pocket costs of care that contribute to material and psychological hardship and can undermine current and future financial security. Latino/Hispanic family caregivers pay nearly double the out-of-pocket costs relative to non-Latino/Hispanic caregivers. CONFIDENCE is a 5-week psychoeducational intervention designed to help caregivers manage out-of-pocket caregiving costs and psychological strain by equipping caregivers with resourcefulness skills and building their self-efficacy in managing care costs. The intervention is culturally tailored to meet the needs of Latino/Hispanic family caregivers. The investigators are conducting a single-arm pre-and post-test trial to determine the preliminary efficacy of the CONFIDENCE intervention among Latino/Hispanic family caregivers to persons living with dementia (NCT05292248). Preliminary results from the first ten study participants, completed as a part of planned interim analyses, indicate a positive change in the hypothesized direction for each study outcome from baseline survey to follow-up surveys, completed within two weeks of program completion. Caregivers who participated in CONFIDENCE reported increased levels of resourcefulness (M=85.1[SD=7.0] vs. M=97.5[SD=5.6], p-value=0.02). We also observed higher levels of caregiver self-efficacy post-intervention (M=40.4[SD=6.0] vs. M=57.2[SD=6.6], p-value<0.001). Lastly, preliminary analyses suggest reduced levels of psychological financial strain following intervention (M=18.3[SD=4.2] vs. M=8.6[SD=2.7], p-value=0.04). Initial results suggest that psychoeducational intervention may prepare caregivers to manage high out-of-pocket care costs. After completing data collection, the study team will examine how intervention participation may affect out-of-pocket caregiving costs. In future studies, we will consider the potential mediating roles of resourcefulness and self-efficacy on study outcomes.

